# Eicosapentaenoic Acid Induces the Inhibition of Adipogenesis by Reducing the Effect of PPARγ Activator and Mediating PKA Activation and Increased COX-2 Expression in 3T3-L1 Cells at the Differentiation Stage

**DOI:** 10.3390/life13081704

**Published:** 2023-08-08

**Authors:** Michael N. N. Nartey, Hidehisa Shimizu, Hikaru Sugiyama, Manami Higa, Pinky Karim Syeda, Kohji Nishimura, Mitsuo Jisaka, Kazushige Yokota

**Affiliations:** 1 Council for Scientific and Industrial Research-Animal Research Institute, Achimota, Accra P.O. Box AH20, Ghana; michaelnartey@csir.org.gh; 2 Estuary Research Center, Shimane University, 1060 Nishikawatsu-cho, Matsue 690-8504, Shimane, Japan; hideshmz@life.shimane-u.ac.jp; 3 Graduate School of Natural Science and Technology, Shimane University, 1060 Nishikawatsu-cho, Matsue 690-8504, Shimane, Japan; n22m813@matsu.shimane-u.ac.jp (H.S.); n23m811@matsu.shimane-u.ac.jp (M.H.); knishimu@life.shimane-u.ac.jp (K.N.); yokotaka@life.shimane-u.ac.jp (K.Y.); 4 Faculty of Life and Environmental Science, Shimane University, 1060 Nishikawatsu-cho, Matsue 690-8504, Shimane, Japan; pinkykarim@life.shimane-u.ac.jp; 5 Interdisciplinary Center for Science Research, Shimane University, 1060 Nishikawatsu-cho, Matsue 690-8504, Shimane, Japan; 6 The United Graduate School of Agricultural Sciences, Tottori University, 4-101 Koyama-Minami, Tottori 680-8553, Tottori, Japan; 7 Institute of Agricultural and Life Sciences, Academic Assembly, Shimane University, 1060 Nishikawatsu-cho, Matsue 690-8504, Shimane, Japan

**Keywords:** n-3 PUFAs, eicosapentaenoic acid, PKA, COX-2, adipogenesis

## Abstract

Obesity has received increasing attention in recent years because it is a factor in the development of non-communicable diseases. The current study aimed to analyze how representative fatty acids (FAs) such as palmitic acid, stearic acid, oleic acid, α-linolenic acid (ALA), and eicosapentaenoic acid (EPA) affected adipogenesis when/if introduced at the differentiation stage of 3T3-L1 cell culture. These FAs are assumed to be potentially relevant to the progression or prevention of obesity. EPA added during the differentiation stage reduced intracellular triacylglycerol (TAG) accumulation, as well as the expression of the established adipocyte-specific marker genes, during the maturation stage. However, no other FAs inhibited intracellular TAG accumulation. Coexistence of Δ^12^-prostaglandin J_2_, a peroxisome proliferator-activated receptor γ activator, with EPA during the differentiation stage partially attenuated the inhibitory effect of EPA on intracellular TAG accumulation. EPA increased cyclooxygenase-2 (COX-2) expression and protein kinase A (PKA) activity at the differentiation stage, which could explain the inhibitory actions of EPA. Taken together, exposure of preadipocytes to EPA only during the differentiation stage may be sufficient to finally reduce the mass of white adipose tissue through increasing COX-2 expression and PKA activity.

## 1. Introduction

Obesity is a factor in the development of non-communicable diseases caused by overnutrition. Previously, obesity was primarily viewed as a concern limited to high-income nations. However, there has been a notable rise in obesity rates in low- and middle-income countries, which previously had lower obesity prevalence [1]. Consequently, obesity has transitioned from being a relatively insignificant public health issue primarily affecting affluent communities to a significant global health threat. It is now recognized as one of the leading contributors to health complications and reduced quality of life. In 2013, an estimated 2 billion individuals worldwide were classified as overweight, with approximately one-third of them categorized as obese. [1]. Obesity is now recognized as a disease and, as noted above, a major public health threat [1]. Since obesity is caused by excess formation of adipose tissue, understanding the mechanisms of adipogenesis is necessary for the prevention of obesity. Functional changes in adipose tissue result from the differentiation of preadipocytes near adipocytes in white adipose tissue into adipocytes. There are three stages in adipogenesis: growth, differentiation, and maturation stages. The mouse preadipogenic 3T3-L1 cell strain is considered a good model to evaluate the three stages in detail, because differentiation of the cells can be easily controlled [2,3]. Typically, 3-isobutyl-1-methylxanthine (IBMX), dexamethasone (Dex), and insulin (Ins) (MDI) are used to cultivate confluent 3T3-L1 preadipocytes. The cells then enter the differentiation stage where clonal expansion is induced, forcing cells into transition from the cell cycle to terminal differentiation. Additionally, the expression of genes required to achieve the phenotype of adipocyte, such as Leptin and Glucose transporter 4 (GLUT4), is provoked [4,5].

The understanding of cAMP’s biological function has increased tremendously since its discovery in the 1960s. cAMP functions primarily by activating exchange proteins directly activated by cAMP (Epac) [6,7] and cAMP-dependent protein kinase A (PKA). PKA is the major target of cAMP. Numerous physiological substrates for PKA have been identified [8], and these substrates can be detected at various sites in the cell. Resting PKA exists as an inactive holoenzyme complex consisting of two regulatory (R) and two catalytic (C) subunits (R_2_C_2_). The R subunit binds to the C subunit to repress its catalytic activity. The binding of cAMP to the R subunit dissociates it from the C subunit, releasing the C subunit to react freely. The effect of cAMP signaling on preadipocyte differentiation has received considerable attention in recent years, because IBMX, an inducer of adipogenesis, has been proven to inhibit a wide-spectrum phosphodiesterase (PDE), which results in increasing intracellular cAMP. For this reason, cAMP was implicated as an inducer of adipogenesis, and PKA, the classical target of cAMP, was also considered to be associated with the differentiation of preadipocytes.

Cyclooxygenase (COX), a prostaglandin (PG) endoperoxide synthase, has two isozymes, COX-1 and COX-2, which are differentially regulated [9]. Studies using adipocytes suggest that changes in COX-2 expression during the differentiation stage of 3T3-L1 cells play a role in the MDI-evoked adipogenesis after the maturation stage [4,5]. Arachidonic acid (AA), which is produced from linoleic acid, one of the dietary n-6 unsaturated fatty acids and liberated from membrane phospholipids by the catalytic activity of phospholipase, is used by COX-2 to biosynthesize PGG_2_. COX-2 further converts PGG_2_ to PGH_2_, an unstable intermediate used as a substrate by certain PG synthases to synthesize bioactive PGs [9]. PGs can be broadly categorized into two main categories: anti-adipogenic and pro-adipogenic PGs. Examples of anti-adipogenic PGs include PGE_2_ [9,10,11,12] and PGF_2α_ [9,13,14]. These PGs inhibit MDI-evoked adipogenesis through the activation of the EP4 receptor and the FP receptor, respectively [9,11,12,13,14]. Pro-adipogenic PGs include PGJ_2_ derivatives, especially Δ^12^-prostaglandin J_2_ (Δ^12^-PGJ_2_), which is a known activator of PPARγ, the master regulator of MDI-evoked adipogenesis [15,16].

Omega-3 oils are polyunsaturated fatty acids (PUFAs) with biological activity, which are determined by the position of the first double bond from the methyl end of the molecule. Among PUFAs, the current study focused on the effect of eicosapentaenoic acid (EPA) on adipogenesis. Regarding the previously reported relationship between EPA and adipogenesis, analyses using mice have shown that dietary supplementation of EPA suppresses adipogenesis through downregulation of FFAR4/GPR120, and further that when EPA is administered at high concentrations, EPA also exerts such effect independently of PPARγ and GPR120-mediated signaling [17,18,19]. In an analysis using 3T3-L1 cells, an increase in β-oxidation elicited by EPA is reported to be related to increased CPT-1 activity [20]. In addition, EPA added during only the growth or only the maturation stage suppresses lipid accumulation during the maturation stage by decreasing PPARγ expression [21,22,23]. However, there are no reports of analyses focusing on the effect of EPA on the differentiation stage of 3T3-L1 cells. Therefore, the current study aimed to determine how EPA added during only the differentiation stage of 3T3-L1 cells impacts adipogenesis evoked by MDI cocktail. In addition, the effect of representative fatty acids (FAs), palmitic acid (PA), stearic acid (SA), oleic acid (OA), and α-linolenic acid (ALA), on MDI-evoked adipogenesis were also confirmed.

## 2. Materials and Methods

### 2.1. Materials

Dex (Cat: D-4902), recombinant human Ins (Cat: 093-06351), fatty acid-free bovine serum albumin (Cat: 011-07493), streptomycin sulfate (Cat: S6501), penicillin G potassium salt (Cat: PENK-10MU), Dulbecco’s modified Eagle medium (DMEM-HEPES) (Cat: D1152-10L), and Oil red O (Cat: 00625-25G) were purchased from Sigma–Aldrich Co. (St. Louis, MO, USA). Wako Pure Chemical Industries Ltd. (Osaka, Japan) supplied IBMX (Cat: 095-03413) and L-ascorbic acid phosphate magnesium salt n-hydrate (Cat: 013-12061). MP Biomedicals (Solon, OH, USA) supplied the fetal bovine serum (FBS) (Cat: 2910154). Arachidonic acid (AA) (Cat: 506-32-1), Δ^12^-PGJ_2_ (Cat: 87893-54-7), H89 (Cat: 130964-39-5), PA (Cat: 57-10-3), SA (Cat: 57-11-4), OA (Cat: 112-80-1), ALA (Cat: 463-40-1), EPA (Cat: 10417-94-4), and forskolin (Cat: 66575-29-9) were acquired from Cayman Chemical (Ann Arbor, MI, USA). Dibutyryl-cAMP (Cat: 16980-89-5) was sourced from Santa Cruz Biotechnology (Dallas, TX, USA). M-MLV reverse transcriptase without ribonuclease H activity (Cat: M3682) was supplied by Promega (Madison, WI, USA). Sigma Genosys Japan (Ishikari, Japan) provided oligonucleotides for the real-time quantitative (RT-q) PCR amplification.

### 2.2. Adipogenesis Induction by 3T3-L1 Cell Culture

Pre-adipogenic mouse 3T3-L1 cells (JCRB9014; JCRB Cell Bank, Osaka, Japan) were cultured following previously established protocols [24,25]. In summary, 3T3-L1 cells were seeded in 35- or 60-mm dishes containing growth medium (GM) at a density of 1 × 10^5^ or 2 × 10^5^, respectively, in the growth stage. The GM consisted of DMEM-HEPES, 10% FBS, penicillin G (100 units/mL), streptomycin sulfate (100 μg/L), and ascorbic acid (200 μM). The dishes were then incubated at 37 °C under 7% CO_2_ until the cells reached confluency. Once the monolayers were confluent, they were exposed to differentiation medium (DM) for 6–48 h. The DM comprised the GM supplemented with Dex (1 μM), IBMX (0.5 mM), and Ins (10 μg/mL), effectively inducing the differentiation of the cells into adipocytes. The cells were further cultured in maturation medium (MM), which consisted of GM supplemented with Ins (5 μg/mL) for 6–10 days. Every 2 days, the MM was replaced with fresh medium to facilitate fat accumulation in the 3T3-L1 cells as they matured. In order to investigate the impact of different agents specifically during the differentiation stage, confluent cell monolayers were incubated in DM supplemented with the respective test compounds for 6–48 h. For a final ethanol concentration of 0.2%, the test substances were dissolved in ethanol and added to the DM.

### 2.3. Measurement of Intracellular Triacylglycerols and Proteins

To quantify intracellular triacylglycerol (TAG) and proteins, the same methods as previously described [24,25] were employed. Cultured mature adipocytes were collected and suspended in phosphate-buffered saline (PBS) without Ca^2+^ and Mg^2+^ (PBS [-]) containing 0.05% trypsin and 0.53 mM EDTA. After that, the suspension was incubated for 5 min at 37 °C. The resultant cell suspensions were next rinsed with PBS (-), split into two halves, and homogenized in 25 mM Tris-HCl buffer (pH 7.4) containing 1 mM EDTA and 1 N NaOH. The portion of the samples homogenized in 25 mM Tris-HCl buffer (pH 7.4) containing 1 mM EDTA were used to quantify the intracellular TAG content, using the Triglyceride E-Test Kits (Cat: 432–40201) (Wako Pure Chemical Industries Ltd., Osaka, Japan). To remove any materials that could interfere with the protein analysis, the proteins in the remaining portion were separated using ice-cold 6% trichloroacetic acid. Then, using fatty acid-free bovine serum albumin as a standard, these proteins were quantified using the Lowry method. The levels of intracellular TAG were normalized to the protein levels and represented in the results as the relative amounts of accumulated TAG.

### 2.4. Visualization of Intracellular Lipids by Oil Red O Staining

MDI-induced differentiated to mature cells were fixed in 10% formalin, and then washed twice with PBS (-). Subsequently, after treating the fixed MDI-induced differentiated to mature cells with 60% isopropanol, these cells were stained by treatment with Oil red O dissolved in 60% isopropanol. The cells treated with Oil red O were then washed with 60% isopropanol, followed by two more washes with PBS (-). Finally, Oil red O-stained intracellular lipids were visualized.

### 2.5. Measurement of Gene Expression Levels

The gene expression levels were quantitatively analyzed following previously established methods [24,25]. In summary, reverse transcription (RT) was performed using M-MLV reverse transcriptase (ribonuclease H activity caused by point mutation), using total RNA (1 g) that was extracted from the cells using acid guanidium thiocyanate/phenol/chloroform after 24 and 48 h after the differentiation stage and on days 6 of the maturation stage. Using oligo-(dT)_15_ (Cat: C1101) and a random 9-mer (Cat: C1181) (Promega Corp., Madison, WI, USA) as primers in the RT process, single-stranded cDNA was synthesized. Using TB Green^TM^ Premix Ex Taq^TM^ II (Tli RNaseH Plus) kits (Cat: RR420A) from Takara Bio Co., Inc., Kusatsu, Japan, and a Thermal Cycler Dice^TM^ Real-Time System and a Thermal Cycler Dice Real Time System III from the same company were utilized to assess the transcript levels by RT-qPCR in accordance with the threshold cycle (CT) and ^ΔΔ^CT techniques specified by the manufacturer. The oligonucleotides used in this study are listed in Table 1. The cycling program included 30 s at 95 °C, 40 cycles at 95 °C for 5 s, and 30 s at 60 °C, followed by 15 s at 95 °C and 30 s at 60 °C. The levels of target gene transcripts were measured and normalized to the levels of *β-Actin*. The target genes’ accession numbers are as follows: *Leptin*, NM_008493; *Glut4*, AB008453; *COX-2*, NM_011198.5; *β-Actin*, NM_007393.

### 2.6. Data Presentation and Statistical Analysis 

The results are presented as mean ± standard deviation (SD). Statistical analysis was performed using Student *t*-tests with Statcel 4 (OMS Publishing Co., Saitama, Japan). The significance level was set at *p* < 0.05.

## 3. Results

### 3.1. Addition of EPA during the Differentiation Stage Effectively Inhibited Adipogenesis Evoked by MDI

**Figure 1:**
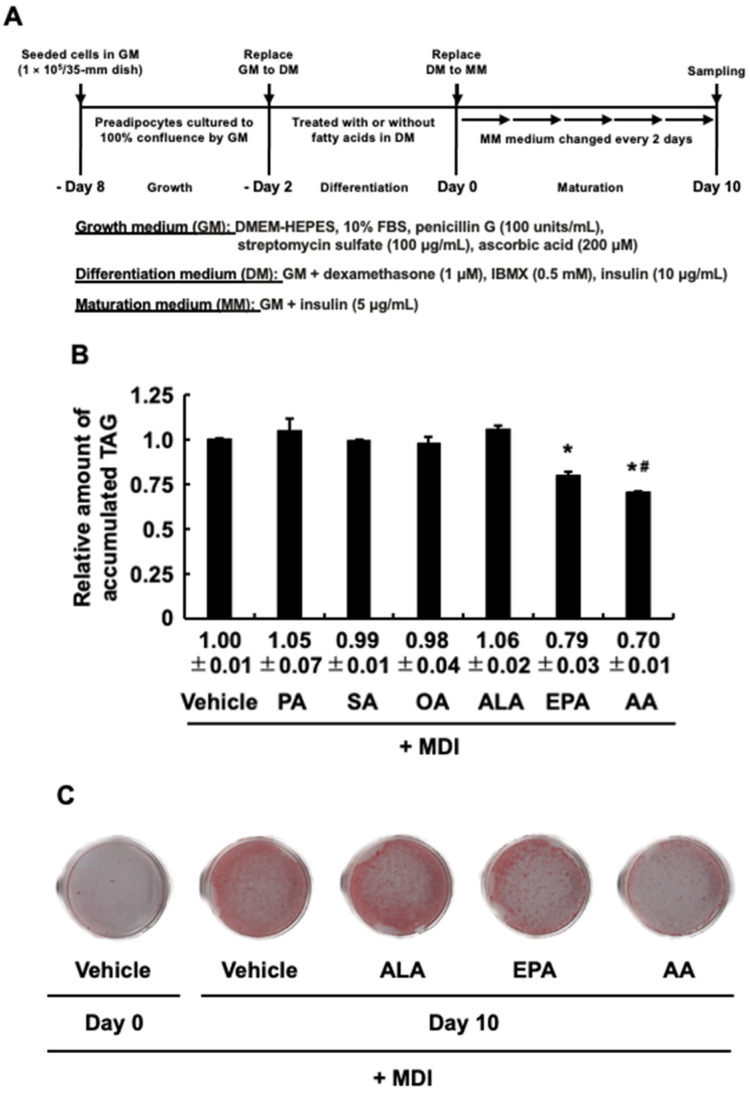
The effects of fatty acids introduced exclusively during the differentiation stage of 3T3-L1 cells on the intracellular triacylglycerol (TAG) accumulation after the maturation stage. (**A**) Experimental protocol. 3T3-L1 cells were incubated with or without palmitic acid (PA) (50 µM), stearic acid (SA) (50 µM), oleic acid (OA) (50 µM), α-linolenic acid (ALA) (50 µM), eicosapentaenoic acid (EPA) (50 µM), or arachidonic acid (AA) (50 µM) during the differentiation stage. (**B**) The intracellular TAG levels after the maturation stage. (**C**) Staining levels of Oil red O at the maturation stage. The data are means ± SD from n = 3, each performed in duplicate. Statistical analysis revealed significant differences compared to the vehicle in DM (* *p* < 0.05) and compared to EPA in DM (# *p* < 0.05). MDI, 3-isobutyl-1-methylxanthine, dexamethasone, and insulin.

We added PA, SA, and OA, which are abundant and the typical fatty acids in red meat, ALA, which is abundant and the typical fatty acid in vegetable fats, and EPA, a metabolite of ALA, which is abundant and the typical fatty acid in fish meat, to 3T3-L1 cells at the differentiation stage and examined their effect on MDI-evoked adipogenesis during the maturation stage as described in Figure 1A. AA was used as a positive control because we and other groups reported that AA added during the differentiation stage suppresses adipogenesis evoked by MDI after the maturation stage [24]. Figure 1B shows that EPA reduced the intracellular TAG accumulation as an indicator of the level of adipogenesis. AA used as a positive control showed similar results to those from EPA, although it accumulated less intracellular TAG than EPA (Figure 1B). Conversely, other fatty acids did not suppress the intracellular TAG accumulation (Figure 1B). To further confirm MDI-evoked adipogenesis from preadipocytes to adipocytes for the results of intracellular TAG accumulation (Figure 1B), visualization of lipids was performed by staining the cells with Oil red O. The lipids were increased on Day 10 as compared to Day 0 (Figure 1C). In addition, when focusing on vehicle, ALA, EPA, and AA, results similar to those obtained with intracellular TAG accumulation were observed in Figure 1B (Figure 1C). In our previous study, adipogenesis evoked by MDI and TAG accumulation were positively correlated [26]. This correlation was also confirmed in the present study, indicating that measuring intracellular TAG accumulation can be used to quantify MDI-evoked adipogenesis. Furthermore, these results indicated that EPA added during the differentiation stage inhibits MDI-evoked adipogenesis as well as AA and that metabolism of ALA to EPA is necessary to show the inhibitory effect. Studies examining the effect of EPA on adipogenesis using 3T3-L1 cells have used a concentration of 100 µM [21,22,23], while anti-inflammatory effects using similar cells have been reported at a concentration of 50 µM [27]. Based on the results of Figure 1B,C, 50 µM EPA added during the differentiation stage was sufficient to be anti-adipogenic besides anti-inflammatory.

### 3.2. Adipocyte-Specific Marker Genes Expression during the Differentiation Stage When 3T3-L1 Cells Are Cultured with EPA or AA

**Figure 2:**
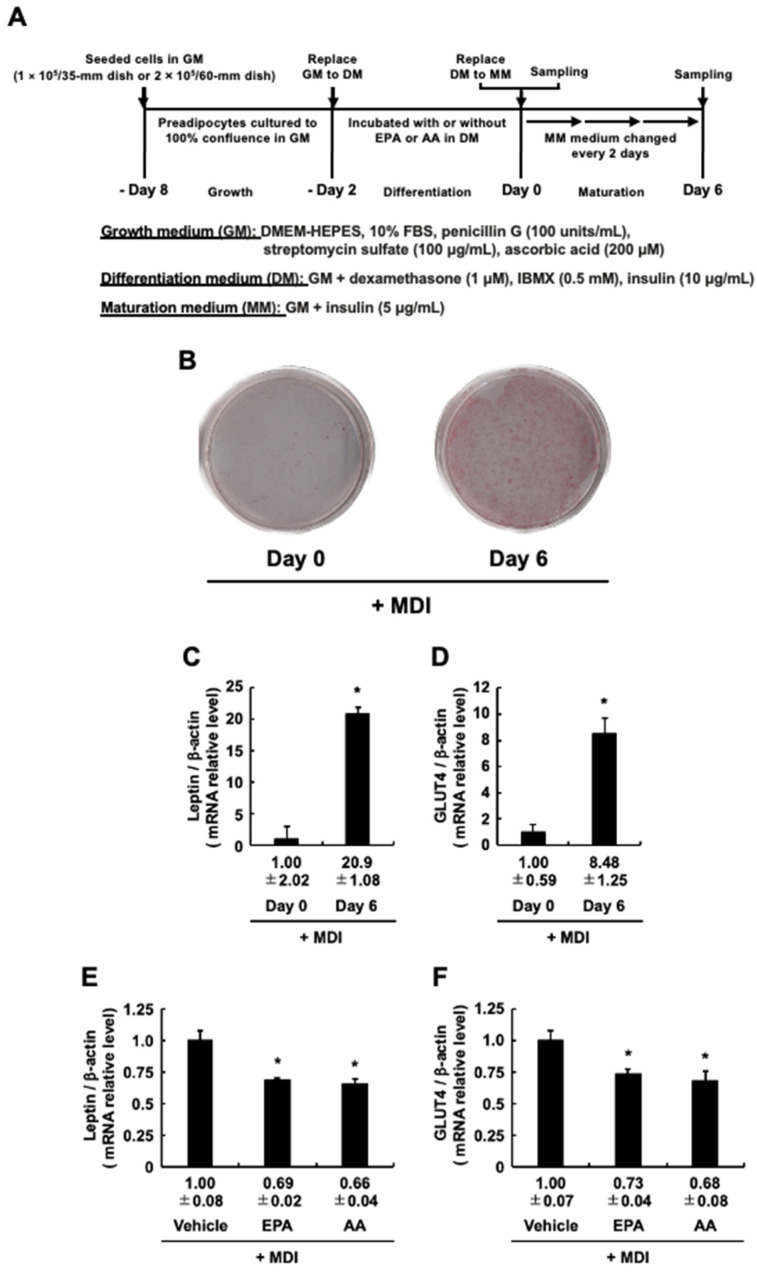
Effects of eicosapentaenoic acid (EPA) or arachidonic acid (AA) added to 3T3-L1 cells exclusively during the differentiation stage on the expression of *Leptin* and *Glut4* genes in the maturation stage. (**A**) Experimental protocol. 3T3-L1 cells were incubated with or without EPA or AA during the differentiation stage. (**B**) Staining levels of Oil red O at the maturation stage. (**C**,**D**) Expression levels of adipogenic marker genes on Day 0 and Day 6. (**E**,**F**) Expression levels of adipogenic marker genes after the maturation stage. The mRNA expression levels for *Leptin* (**C**,**E**) and *Glut4* (**D**,**F**) were determined. The data are presented as means ± SD from n = 4 for (**C**,**D**), and from n = 3 for (**E**,**F**). Statistical analysis revealed significant differences compared to the vehicle (control) in DM (* *p* < 0.05). MDI, 3-isobutyl-1-methylxanthine, dexamethasone, and insulin.

In our experimental system, MDI-evoked adipogenesis is fully induced on Day 6, and the expression levels of adipogenic differentiation marker genes, *Leptin* and *Glut4*, two established adipocyte-specific marker genes, are increased accordingly [4,5]. Based on the results in Figure 1, the mRNA expression levels of *Leptin* and *Glut4* were further analyzed, as described in Figure 2A. The present study also confirmed that the staining with Oil red O and the mRNA expression levels of these two genes were significantly increased on Day 6 compared to Day 0 (Figure 2B–D). That is, induction of MDI-evoked adipogenesis occurred without problems on day 6. Under this experimental condition, the mRNA expression levels of these two genes were predictably downregulated by EPA as well as by AA [24], the positive control, added during the differentiation stage (Figure 2E,F). Together with the results in Figure 1, these findings suggest that the addition of EPA at the differentiation stage, as well as AA, reduced the intracellular TAG accumulation due to the suppression of MDI-evoked differentiation into mature adipocytes.

### 3.3. Δ^12^-PGJ_2_, a PPARγ Activator, Partially Attenuates the Anti-Adipogenic Effect of EPA

**Figure 3:**
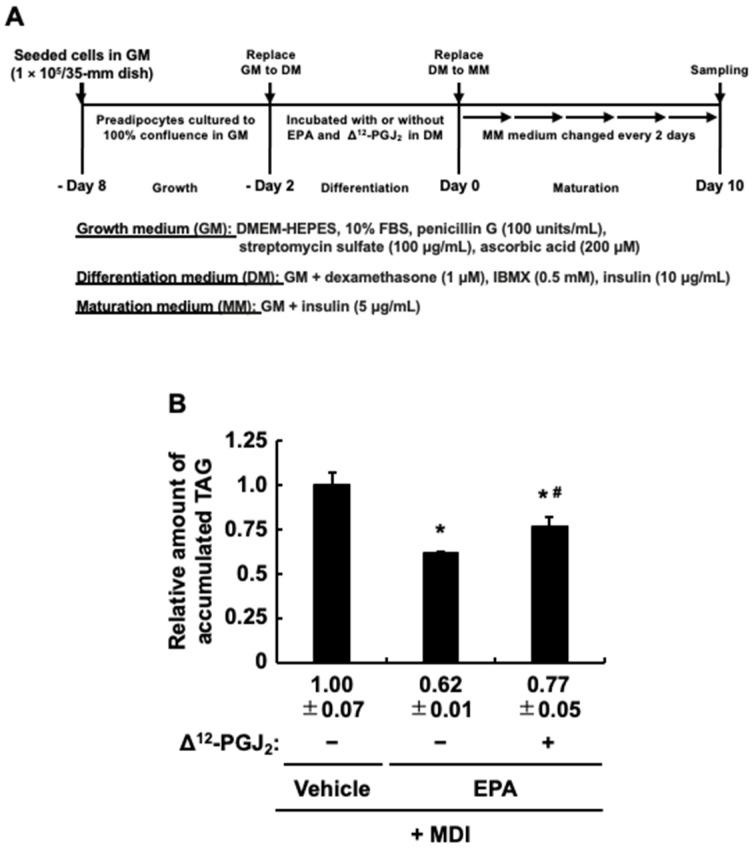
The effects of Δ^12^-prostaglandin J_2_ (Δ^12^-PGJ_2_) on the suppression of the intracellular triacylglycerol (TAG) accumulation in 3T3-L1 cells treated with eicosapentaenoic acid (EPA) exclusively during the differentiation stage. (**A**) Experimental protocol: 3T3-L1cells were incubated with or without Δ^12^-PGJ_2_ (1 µM), or EPA (50 µM) during the differentiation stage. (**B**) The intracellular TAG levels after the maturation stage. The data are presented as means ± SD from n = 3, each per-formed in duplicate. Statistical analysis revealed significant differences compared to the vehicle in DM (* *p* < 0.05) and compared to EPA in DM (# *p* < 0.05). MDI, 3-isobutyl-1-methylxanthine, dexamethasone, and insulin.

Because PPARγ is a typical master regulator involved in the induction of adipogenesis [9] and EPA can function as an activator of PPARγ [28,29], the anti-adipogenic effect of EPA added during the differentiation stage was slightly weaker than that of AA as expected (Figure 1B). In addition to these reports and results, since we previously confirmed that Δ^12^-PGJ_2_, a typical PPARγ activator, added at the differentiation stage increases intracellular TAG buildup in MDI-evoked mature adipocytes [24], we assumed that the coexistence of EPA and Δ^12^-PGJ_2_ during the differentiation stage would further weaken the anti-adipogenic effect of EPA, and was analyzed as described in Figure 3A. Based on the results in Figure 1B, intracellular TAG accumulation was measured as an indicator of MDI-evoked adipogenesis. The coexistence of Δ^12^-PGJ_2_ and EPA partially but significantly suppressed the anti-adipogenic effect of EPA, but as expected, the coexistence of Δ^12^-PGJ_2_ and EPA attenuated the anti-adipogenic effect of EPA compared to that of EPA alone (Figure 3B). These results suggested that EPA has both anti-adipogenic and PPARγ-mediated pro-adipogenic effects, while the anti-adipogenic effect is stronger, resulting in reduced intracellular TAG accumulation in MDI-evoked mature adipocytes.

### 3.4. The Impact of Protein Kinase A (PKA) Activation on the Effect of EPA Added Exclusively during the Differentiation Stage of 3T3-L1 Cells on Adipogenesis Evoked by the MDI Cocktail

**Figure 4:**
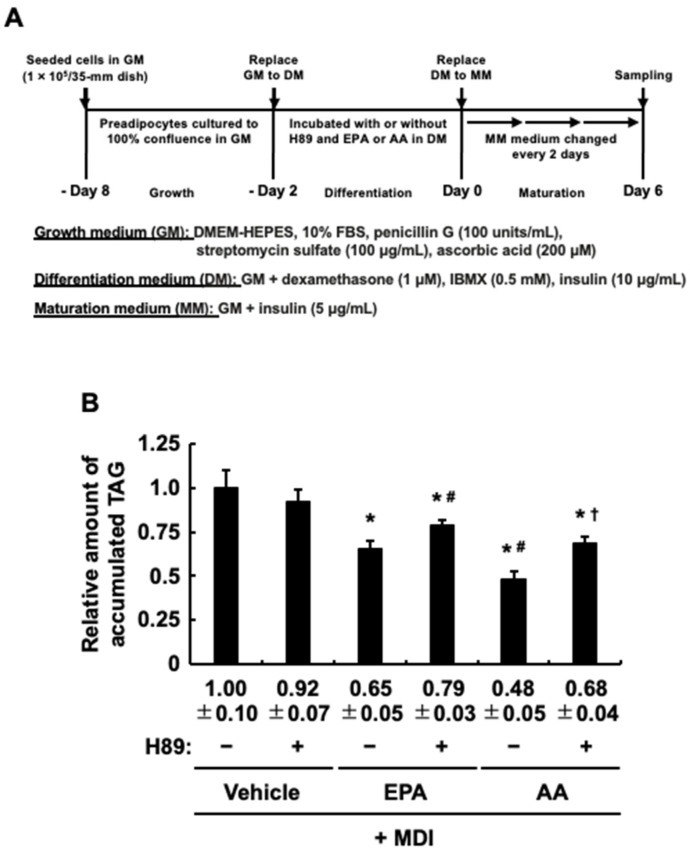
Effects of H89 activation on the suppression of the intracellular triacylglycerol (TAG) accumulation in 3T3-L1 cells incubated with eicosapentaenoic acid (EPA) or arachi-donic acid (AA) at the differentiation stage. (**A**) Experimental protocol. 3T3-L1cells were incubated with or without H89 (10 µM), EPA (50 µM) or AA (50 µM) during the differentiation stage. (**B**) The intracellular TAG levels after the maturation stage. The data are presented as means ± SD from n = 3, each performed in duplicate. Statistical analysis showed significant difference compared with vehicle in DM (* *p* < 0.05), compared with EPA in DM (# *p* < 0.05), and compared with AA in DM († *p* < 0.05). MDI, 3-isobutyl-1-methylxanthine, dexamethasone, and insulin.

**Figure 5:**
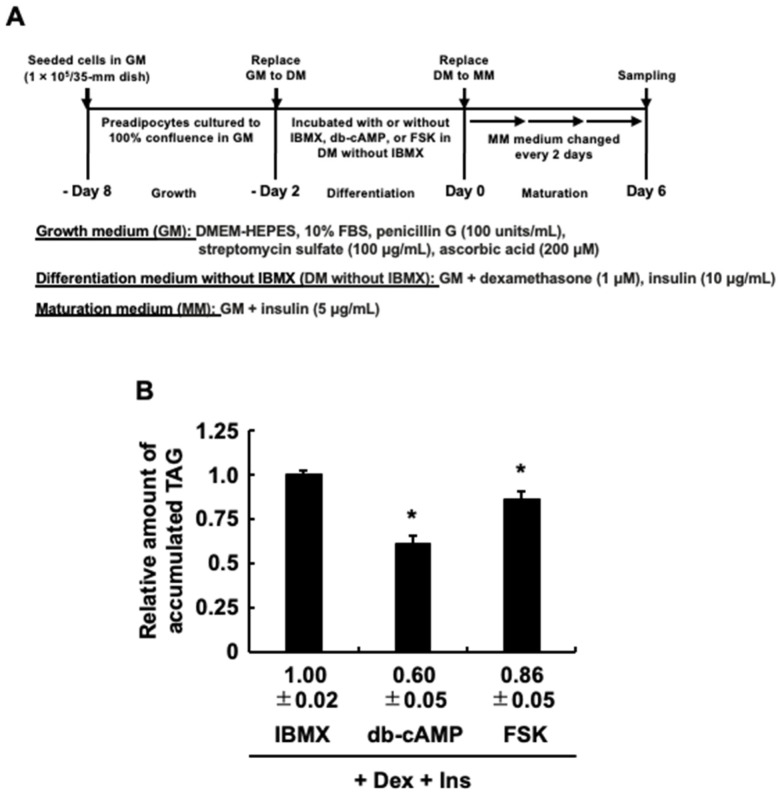
The effects of activating protein kinase A (PKA) at the differentiation stage on the in-tracellular triacylglycerol (TAG) accumulation at the maturation stage of 3T3-L1 cells. (**A**) Experimental protocol. 3T3-L1 cells were incubated with or without IBMX (0.5 mM), dibutyryl-cAMP (db-cAMP) (100 µM), or forskolin (FSK) (10 µM) during the differentiation stage. (**B**) The intracellular TAG levels after the maturation stage. The data are presented as means ± SD from n = 3, each performed in duplicate. Statistical analysis revealed significant differences compared to IBMX (* *p* < 0.05). MDI, 3-isobutyl-1-methylxanthine, dexamethasone, and insulin.

Since EPA so far exhibited anti-adipogenic effects similar to those of AA, we tried, following the procedures in Figure 4A, to determine whether EPA exerts the same PKA-mediated anti-adipogenic effect as AA [30,31]. Figure 4B shows that, as reported previously, the addition of H89, a general PKA inhibitor, with AA during the differentiation stage partially canceled the inhibitory effect of AA on the MDI-evoked TAG accumulation [30,31]. The inhibitory effect of EPA was also partially canceled by H89 (Figure 4B). These results indicated that activation of PKA induced by EPA, as well as AA, inhibits MDI-evoked adipogenesis. Based on these results, to further confirm whether activation of PKA induces the anti-adipogenic effect, dibutyryl-cAMP or forskolin, which both induce activation of PKA, was treated and analyzed instead of IBMX in DM, and the intracellular TAG levels were analyzed (Figure 5A). Figure 5B demonstrates that 3T3-L1 cell culture with dibutyryl-cAMP or forskolin at the differentiation stage effectively suppresses the adipogenesis evoked by MDI after the maturation stage. Taken together, the activation of PKA may be one of the key molecules involved in the repression of MDI-evoked adipogenesis by EPA and AA during the maturation stage.

### 3.5. EPA-Induced COX-2 Expression during the Differentiation Stage

**Figure 6:**
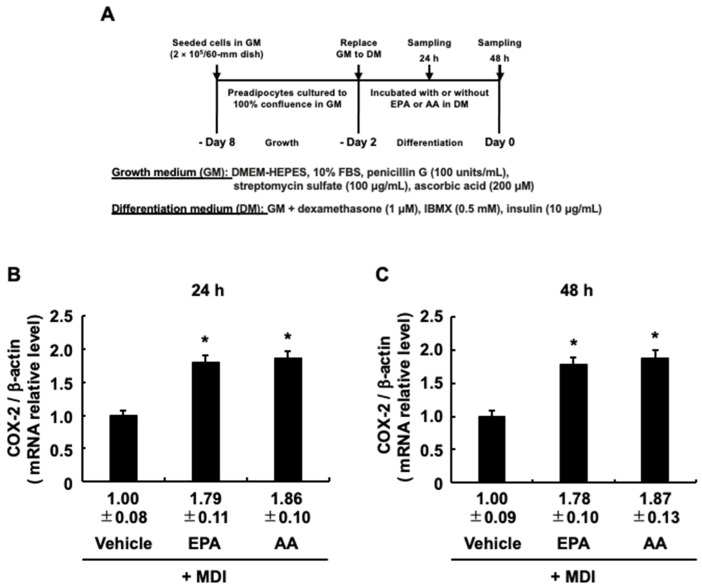
The effects of administration of 3T3-L1 cells with eicosapentaenoic acid (EPA) or ara-chidonic acid (AA) on the expression of *COX-2* during the differentiation stage. (**A**) Experimental protocol. 3T3-L1 cells were incubated with or without EPA (50 µM) or AA (50 µM) during the differentiation stage. (**B**,**C**) Expression levels of *COX-2* during the differentiation stage. The mRNA expression levels for *COX-2* at 24 hours (**B**) and 48 hours (**C**) upon initiating the differentiation stage were determined. The data are presented as means ± SD from n = 3. Statistical analysis revealed significant differences compared to the vehicle (control) in DM (* *p* < 0.05). MDI, 3-isobutyl-1-methylxanthine, dexamethasone, and insulin.

Activation of PKA is associated with increased *COX-2* expression elicited by AA added at the differentiation stage [30,31]. Therefore, EPA, like AA, added during the differentiation stage may also lead to increased COX-2 expression. To test this hypothesis, we followed the procedure in Figure 6A and found that EPA added during the differentiation stage led to increased *COX-2* mRNA expression as well as AA (Figure 6B,C). Our previous study analyzing overexpressed COX-2 or its antisense revealed that COX-2 is involved in suppressing MDI-evoked adipogenesis during the maturation stage [4,5]. Based on these reports, the anti-adipogenic effect of EPA, as well as AA, may be due to the increased *COX-2* expression.

## 4. Discussion

**Figure 7:**
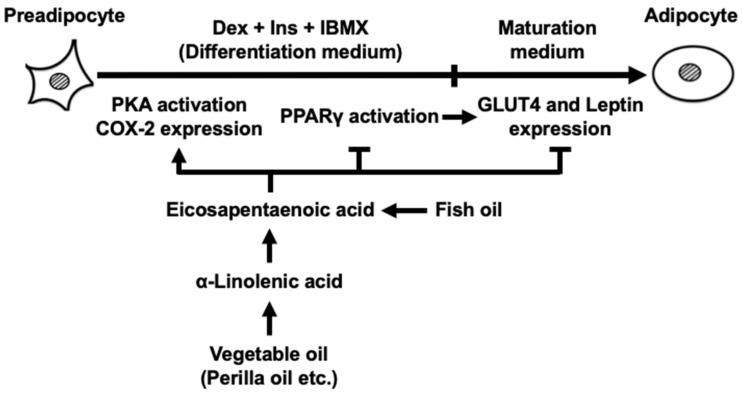
This schematic illustrates the role of eicosapentaenoic acid (EPA) specifically during the differentiation stage of 3T3-L1 cells and the predicted mechanisms by which EPA suppresses adipogenesis evoked by IBMX, Dex, and Ins (MDI). EPA induced an activation of PKA and an increase in COX-2 expression as well as a suppression of PPARγ activation in the differentiation stage, which may eventually inhibit MDI-evoked adipogenesis during the maturation stage. COX-2, cyclooxygenase-2; PKA, protein kinase A; Dex, dexamethasone; Ins, insulin; IBMX, 3-isobutyl-1-methylxanthine; PPARγ, peroxisome proliferator-activated receptor-γ; GLUT4, glucose transporter 4.

The current findings are summarized in Figure 7. The novel findings in the current study are that exposing 3T3-L cells to EPA at only the differentiation stage suppressed MDI-evoked adipogenesis during the maturation stage with a decrease in intracellular TAG accumulation and expression levels of the established adipocyte-specific marker genes. In addition, when EPA coexisted with Δ^12^-PGJ_2_, a PPARγ activator, the anti-adipogenic effect of EPA was partially attenuated by Δ^12^-PGJ_2_. Furthermore, activation of PKA elicited by EPA at the differentiation stage may contribute to the anti-adipogenic effect. Taken together, in addition to increased *COX-2* expression, the effect of EPA on the inhibitory actions of PPARγ activator and activation of PKA elicited by EPA at the differentiation stage may have significant implications in the inhibition of MDI-evoked adipogenesis during the maturation stage.

The current study shows that ALA did not exhibit an anti-adipogenic effect like EPA, indicating that ALA must be metabolized to EPA to exert anti-adipogenic effect. However, when rats were fed perilla, which is rich in ALA and accounts for 50% of the total fatty acid content [32,33], plasma EPA concentrations in rats were elevated [34,35], suggesting that ALA intake also exerts an anti-adipogenic effect via metabolism to EPA in the body. Therefore, in regions where seafood rich in EPA is hard to obtain, ingestion of vegetable oil, such as perilla oil, can exert the anti-obesity effect of EPA in the body. Furthermore, when inclusion complexes of perilla oil with γ-cyclodextrin were ingested by rats, the amount of ALA absorbed into the body increased [34,35]. That is, the ingestion of inclusion complexes of perilla oil with γ-cyclodextrin will have a greater anti-obesity effect than the ingestion of ALA alone because the inclusion complex of perilla oil with γ-cyclodextrin can be expected to produce more EPA in the body. However, this report did not analyze the ingestion of inclusion complexes of perilla oil with γ-cyclodextrin in an obese model; future reports are expected.

Leptin is reported to decrease the amount of intracellular TAG accumulated in 3T3-L1 cells, although it does not affect their proliferation or differentiation [36]. Based on this report, EPA added during the differentiation stage is speculated to increase intracellular TAG accumulation during the maturation stage because it reduces *Leptin* expression during the maturation stage. However, the results of the current study show the opposite; while decreasing *Leptin* expression, EPA added during the differentiation stage also decreased the intracellular TAG buildup throughout the maturation stage. Since *Leptin* is a marker of adipocyte differentiation [4,5], the decrease in *Leptin* expression during the maturation stage implies the suppression of MDI-evoked adipogenesis by EPA added at the differentiation stage. In addition, the current study also shows that the suppression of *Glut4* expression at the maturation stage was elicited by EPA added at the differentiation stage. Similarly, since *Glut4* is a marker of adipogenic differentiation [4,5], decreased *Glut4* expression indicates that MDI-evoked adipogenesis was suppressed by EPA added during the differentiation stage. Furthermore, we consider that the suppression of intracellular TAG buildup throughout the maturation stage may be due to the suppression of glucose-derived intracellular TAG synthesis associated with the decreased *Glut4* expression elicited by EPA added during the differentiation stage.

Even though IBMX, required for differentiation into adipocytes, is a cAMP-elevating agent, the current study demonstrated that the coexistence of H89 with EPA during the differentiation stage partially cancelled the anti-adipogenic effect of EPA. That is, EPA-induced activation of PKA mediated by cAMP was suggested to play a role in the inhibition of MDI-evoked adipogenesis during the maturation stage. At least one reason for this is that adipogenesis evoked by MDI requires activation of both exchange proteins, Epac and PKA, which are directly activated by increased cAMP production during the differentiation stage [37,38], while the activation of PKA alone at the differentiation stage is reported to inhibit adipogenesis evoked by MDI [39]. In fact, the current analysis using dibutyryl-cAMP and forskolin once again confirmed that the activation of PKA alone at the differentiation stage inhibited MDI-evoked adipogenesis. Therefore, cAMP expected to be produced intracellularly by EPA added during the differentiation stage may activate PKA.

As noted above, PKA activated by EPA during the differentiation stage may be involved in the repression of MDI-evoked adipogenesis throughout the maturation stage, but it is unclear how EPA activated PKA. One possibility is that EPA binds to G protein-coupled receptors (GPCRs) and activates PKA. In fact, EPA is reported to activate PKA in rat ventricular myocytes [40]. A previously reported GPCR of EPA expressed in 3T3-L1 adipocytes is FFAR4/GPR120 [41]. However, the Gα subunit of this GPCR is Gi, which inhibits the activation of adenylate cyclase [42]. Therefore, it is possible that EPA does not activate PKA, but that EPA metabolites such as resolvins activate PKA and suppress adipogenesis. Another possibility is the action of anti-adipogenic PGs, PGE_2_ and PGF_2α_, AA metabolites produced by COX-2, whose expression is increased by EPA. Based on analysis of stably overexpressed COX-2 or its antisense at the differentiation stage of 3T3-L1 cells, we found that COX-2 exerts an anti-adipogenic effect by mediating the production of PGE_2_ and PGF_2α_ [4,5]. In addition, activation of PKA is considered to be associated with the anti-adipogenic effect of PGE_2_ [11]. The current study shows that EPA led to increased *COX-2* expression and H89 suppressed the anti-adipogenic effect elicited by EPA, suggesting that PGE_2_ generated via COX-2 from endogenous AA may act on the anti-adipogenic effect elicited by EPA. The fact that H89 did not completely suppress the anti-adipogenic effect elicited by EPA may be due to the residual effect of PGF_2α_ produced via COX-2 from endogenous AA [24,31,43]. In any case, further analysis is needed to determine whether its anti-fat effect is a direct effect of EPA or an effect of metabolites of EPA and AA.

Since Ins with EPA coexist at the differentiation stage in the current study, and Ins signaling is also required for adipogenesis, in our investigation, we directed our attention towards Ins signaling as a potential mechanism through which the activation of PKA, facilitated by EPA, exerts its suppressive effects on MDI-evoked adipogenesis during the maturation stage. Upon binding to the insulin receptor (IR), Ins triggers the phosphorylation of tyrosine residues on both the IR and its substrate, insulin receptor substrate-1 (IRS-1) [44,45]. Consequently, this initiates a signaling cascade involving phosphatidylinositol 3-kinase and Akt, ultimately leading to the differentiation of preadipocytes into adipocytes [46]. On the other hand, IRS-1 is reported to be phosphorylated at not only tyrosine residues but also serine residues [39,47,48,49]. PKA activated by forskolin directly binds to IRS-1 and phosphorylates its serine789 [39]. This phosphorylation of IRS-1 inhibits Ins-induced IRS-1 tyrosine phosphorylation [39]. Since Ins plays an important role in MDI-evoked adipogenesis [50], cAMP-mediated activation of PKA is shown to inhibit MDI-evoked adipogenesis during the maturation stage by modulating Ins signaling through inactivation by serine phosphorylation of IRS-1 [39]. Thus, EPA added at the differentiation stage may also impede MDI-evoked adipogenesis at the maturation stage by suppressing Ins signaling through a similar mechanism.

Although EPA and Δ^12^-PGJ_2_, a pro-adipogenic PG, are both activators of PPARγ, a master regulator of MDI-evoked adipogenesis, the coexistence of EPA and Δ^12^-PGJ_2_ during the differentiation stage in the current study suppressed MDI-evoked adipogenesis during the maturation stage. This result is similar to that of AA used as a positive control [24], suggesting that increased PGF_2α_ and PGE_2_ production with increased *COX-2* expression is associated with the anti-adipogenic effect of EPA, as is the mechanism observed in AA [24]. In addition, as already mentioned above, based on previous validations using overexpressed COX-2 or its antisense, COX-2-mediated PGF_2α_ and PGE_2_ production may play a significant role in suppressing adipogenesis [4,5]. PGF_2α_ and PGE_2_ are indicated to exert their anti-adipogenic effects by binding to specific FP and EP4 receptors, respectively [11,12,13,14]. Stimulation of the FP receptor by PGF_2α_ triggers the reduction in the PPARγ transcriptional activity by promoting its phosphorylation through the activation of extracellular signal-regulated kinase, thereby inducing the anti-adipogenic effect [51]. Activation of the EP4 receptor by PGE_2_ produces PGF_2α_ by increasing COX-2 expression through activation of cyclic AMP response element-binding protein [52]. That is, the activation of the EP4 receptor promotes the production of PGF_2α_, which subsequently diminishes the PPARγ transcriptional activity by activating the FP receptor. Consequently, the increased production of PGE_2_ at the differentiation stage exerts a similar mechanism as PGF_2α_, thereby suppressing MDI-evoked adipogenesis at the maturation stage. If EPA-mediated increase in *COX-2* expression nullifies the action of PPARγ activators by the above mechanisms, it could be an important factor leading to its anti-adipogenic effect. However, the above action mechanisms of EPA on anti-adipogenic effect are only speculative, and thus further studies are needed.

## 5. Conclusions

The results of the current study indicate that EPA suppresses adipogenesis not only when added during the proliferation and maturation stages, as previously reported, but also when added during only the differentiation stage. Therefore, the reduction in white adipose tissue volume elicited by EPA intake is predicted to be the result of the anti-adipogenic effect of EPA in all adipogenic processes during the proliferation, differentiation, and maturation stages. Furthermore, the anti-adipogenic effect of ALA intake would require at least some metabolism to EPA.

## Figures and Tables

**Table 1:** Forward (F) and reverse (R) primers for target genes.

GenBank Accession No.	Target Genes	Primers (5→3′)	Length (bp)	Tm (°C)	Product Length (bp)
NM_008493	*Leptin*	F: TTTCACACACGCAGTCGGTA	20	59.90	149
		R: CACATTTTGGGAAGGCAGGC	20	60.04	
AB008453	*Glut4*	F: GGATTCCATCCCACAAGGCA	20	60.03	158
		R: CCAACACGGCCAAGACATTG	20	60.04	
NM_011198.5	*COX-2*	F: GTTTGTTGAGTCATTCACCAG	21	55.36	224
		R: TGTAGAGGGCTTTCAATTCTG	21	55.59	
NM_007393.5	*β-Actin*	F: GCGGGCGACGATGCT	15	59.84	197
		R: TGCCAGATCTTCTCCATGTCG	21	59.86	

## Data Availability

The data presented in this study are available on request from the corresponding author.
